# P-80. The microbiology of periprosthetic joint infections as revealed by sonicate cultures in Korea: Routine use of fungal and mycobacterial cultures is necessary?

**DOI:** 10.1093/ofid/ofae631.287

**Published:** 2025-01-29

**Authors:** Kyung-Hwa Park, Hae Sung Jeong, Sung un Shin, Uh Jin Kim, Seong Eun Kim, Seung-ji Kang, Sook In Jung

**Affiliations:** Chonnam National University Medical School, GwangJu, Kwangju-jikhalsi, Republic of Korea; Department of Infectious Diseases, Chonnam National University Medical School,Gwangju, Korea., Gwangju, Kwangju-jikhalsi, Republic of Korea; Chonnam National University hospital, Kangju, Kwangju-jikhalsi, Republic of Korea; Chonnam National University Medical School, GwangJu, Kwangju-jikhalsi, Republic of Korea; Chonnam National University Medical School, GwangJu, Kwangju-jikhalsi, Republic of Korea; Chonnam National University Medical School, GwangJu, Kwangju-jikhalsi, Republic of Korea; Chonnam National University Medical School, GwangJu, Kwangju-jikhalsi, Republic of Korea

## Abstract

**Background:**

Although sonication is a valuable diagnostic tool for periprosthetic joint infections (PJI), it is not commonly utilized. We analyzed sonicate and intraoperative tissue culture results obtained from three hospitals to define the microbial etiology of PJIs in Korea. Furthermore, we investigated necessity of conducting regular fungal and mycobacterial cultures.

**Methods:**

We retrospectively analyzed data for patients with suspected orthopedic-related infections between 2017 and 2022, who had undergone prostheses removal surgery. We included 193 patients with suspected PJIs, and bacterial (n = 193), fungal (n = 193), and mycobacterial (n = 186) cultures were conducted on both sonicate and intraoperative tissue samples. The diagnosis of PJI was based on the European Bone and Joint Infection Society (EBJIS) criteria.

**Results:**

Out of 193 patients, 121 (62.7%) had positive sonicate cultures, while 112 (58.0%) had positive periprosthetic tissue cultures. According to EBJIS criteria, a total of 181 patients were diagnosed with PJI, and 141 patients received microbiological confirmation through sonicate fluid culture or tissue culture. Of the 181 patients, 28 were classified with acute PJI (within 3 months of implantation) and 153 with chronic PJI. Among 141 patients, *staphylococci* were the most common organisms, accounting for 51.8% of cases, followed by Gram-negative organisms (15.6%), fungus (8.5%), and mycobacteria (3.5%). Nearly 91.7% of fungal isolates were *Candida* species, which also grew in bacterial cultures. In total, 11 cases cultured positive only in tissue culture, whereas 20 cases cultured positive only in sonicate culture. The antibiotic treatment plans were adjusted according to culture results.

**Conclusion:**

Utilizing sonicate culture has greatly assisted in identifying pathogens responsible for chronic indolent PJIs, allowing suitable antimicrobial treatment. Based on few cases involving non-*Candida* and mycobacterial infections, it appears that routine fungal and mycobacterial cultures may not be necessary.

**Disclosures:**

**All Authors**: No reported disclosures

